# Inter-outbreak stability reflects the size of the susceptible pool and forecasts magnitudes of seasonal epidemics

**DOI:** 10.1038/s41467-019-10099-y

**Published:** 2019-05-30

**Authors:** Martin Rypdal, George Sugihara

**Affiliations:** 1 Department of Mathematics and Statistics, UiT—The Arctic University of Norway, Tromsø, 9019 Norway; 2 Scripps Institution of Oceanography, University of California San Diego, 9500 Gilman Drive, La Jolla, CA 92093-0202 USA

**Keywords:** Ecological epidemiology, Infectious diseases

## Abstract

For dengue fever and other seasonal epidemics we show how the stability of the preceding inter-outbreak period can predict subsequent total outbreak magnitude, and that a feasible stability metric can be computed from incidence data alone. As an observable of a dynamical system, incidence data contains information about the underlying mechanisms: climatic drivers, changing serotype pools, the ecology of the vector populations, and evolving viral strains. We present mathematical arguments to suggest a connection between stability measured in incidence data during the inter-outbreak period and the size of the effective susceptible population. The method is illustrated with an analysis of dengue incidence in San Juan, Puerto Rico, where forecasts can be made as early as three to four months ahead of an outbreak. These results have immediate significance for public health planning, and can be used in combination with existing forecasting methods and more comprehensive dengue models.

## Abstract (web summary)

Directly measuring the size of the susceptible population is usually unfeasible before dengue outbreaks. Here, the authors show that the stability of low-incidence periods provides a proxy measure, which can be estimated from incidence data, and show its utility for forecasting outbreaks.

## Introduction

Dengue is a systemic viral infection with an estimated 390 million human cases per year, worldwide^1^. The four currently known dengue virus serotypes (DENV1−4) are transmitted between humans via the Aedes aegypti and Aedes albopictus mosquitoes^2^. Emerging work on dengue epidemic outbreaks reveals a complex problem involving antigenic differences between and within serotypes, changes in human populations caused by immune adaptation and migration patterns, complex mosquito vector ecology, climate effects, changing transmissivity and viral evolution among others^3–11^. However desirable as a scientific tool, an explicit and highly detailed model that includes such factors would have limited utility as a public health tool because of the difficult information it would require as inputs. Notwithstanding, when viewed as a dynamic systems problem such mechanistic information may already be contained and potentially accessible in other more readily obtainable observables such as incidence data^12,13^. Although the epidemiological variable most targeted and most readily measured is the infection rate, the size of the susceptible pool (the total number of individuals at risk of infection) can also be important for determining outbreak magnitude, but directly measuring it is problematic and may not be feasible in advance of an outbreak. Indeed existing methods for susceptible reconstruction^7,14–16^ requires information that prohibits use in forecasting.

Here we provide theoretical and empirical evidence to show that information about susceptibles that cannot be observed directly can be captured by a dynamic proxy variable that can be calculated in real time from incidence data alone. Specifically, we show that the stability of low-disease periods, when the dynamics are most sensitive to small variations, predicts the magnitude of the ensuing outbreak. We demonstrate the method using incidence data on dengue epidemics in San Juan Puerto Rico, to show how it can predict both the peak and the cumulative magnitude of an outbreak, and at the same time provide a quantitative early-warning indicator to identify its onset. Such forecasts can be made months in advance and as such are potentially useful for informing public health initiatives in planning and resource allocation.

## Results

### Computing a proxy for susceptibles

**Fig. 1:**
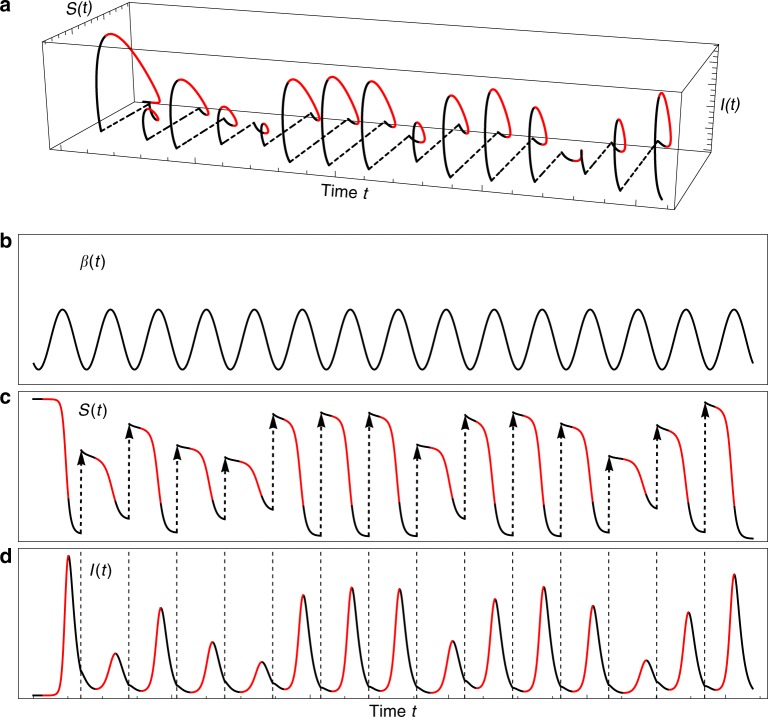
Schematic illustration of SIR dynamics with randomly reset susceptible populations. **a** Schematic illustration showing an attractor expanded out in time with unstable outbreak periods (red) and stable inter-outbreak periods (black) that are stochastically reset (dashed lines). The attractor was constructed from a realization of an SIR model with a periodically varying *β*(*t*) to represent the seasonal cycle, as shown in (**b**), but where each year the population’s susceptible pool is drawn randomly (the stochastic reset). **c** The annually reset dynamics of *S*(*t*) is shown in (**c**). **d** The dynamics of *I*(*t*). The figure is constructed using *β*(*t*) = *a* + *b*(1 − cos(2*πt*/*τ* − *ϕ*)), and parameters *τ* = 1 year, *a* = 0.0005, and *b* = 0.001. At times *t* = *kτ*, *k* = 1, …, 14, the initial conditions were reset to *S* = *r*, where *r* is a random variable with the uniform distribution over {60, 70, …, 120}

**Fig. 2:**
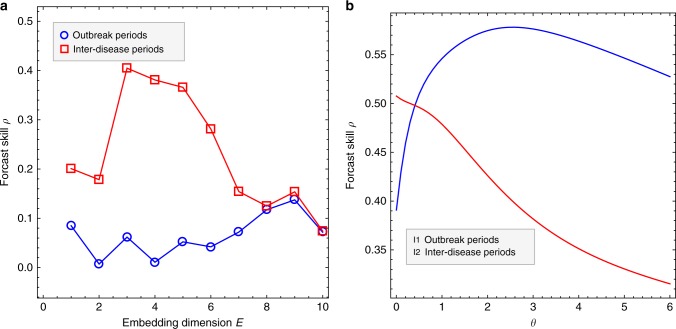
Empirical dynamical analysis for inter-disease periods and outbreak periods in the time series of dengue incidence in San Juan. **a** The low optimal embedding dimension^21^ of the inter-outbreak period (*E* = 3) is consistent with contraction of the dynamics onto stable states, while the outbreak period is higher dimensional (*E* = 9). **b** The S-map test for nonlinearity^20^ shows linear equilibrium dynamics for the inter-disease periods and nonlinear dynamics for the outbreak periods, consistent with the hypothesized mixed model set up (see Methods)

The theoretical set up is illustrated in Fig. 1 where the attractor (composed of annual epidemic cycles) is portrayed as having trajectories with a discontinuity at the end of each outbreak. Each annual excursion has two parts: an inter-disease period and the outbreak itself. This suggests a mixed modeling approach where we distinguish the equilibrium dynamics of the inter-outbreak period (April-August for dengue in San Juan) from the ensuing unstable outbreak dynamics. This distinction is supported by an EDM analysis of dengue in Fig. 2 (see Methods). Importantly, we view the transition from the previous year’s outbreak to the early inter-outbreak period as a stochastic discontinuity in the otherwise conitnuous dynamics. It is a time when complex vital dynamic mechanisms set the initial conditions that characterize the susceptible pool for the next outbreak— a high dimensional gap in the temporal evolution. In simple terms, this corresponds to a class of models having local disease-free equilibria that are not unique and are not endemic, but are parameterized anew with each epidemic cycle.  During the inter-outbreak period the attractor is trivial (a stable disease-free equilibrium), higher dimensional during the outbreak itself, and very high dimensional (stochastic discontinuity) between cycles (a hypothesis validated for dengue in Fig. 2). Again, the discontinuity determines which inter-outbreak trajectory the system will follow into the next epidemic cycle, and thereby sets the initial conditions for the ensuing outbreak.

As a specific simple example, note that in the basic SIR model (where *I* = infected and *S* = susceptible, *R* = recovered, and *β* and *γ* are phenomenological parameters that relate to the force of infection and the basic reproduction number),

1 $$\begin{array}{l}\dot I = \beta SI - \gamma I\\ \dot S = - \beta SI\\ \dot R = \gamma I\end{array}$$

any state with *I* = 0 is an equilibrium point of the system independent of the value *S*. Thus, when the system is close to a disease-free equilibrium, *S* = S* becomes a system parameter, and the linearized equation for the fluctuations around the equilibrium becomes $\dot I = (\beta S^ \ast - \gamma )I$. Because, the relation between the effective susceptible pool and the leading eigenvalue is

2 $$\lambda = \beta S^ \ast - \gamma ,$$

*λ* is a proxy for S*. Thus, the stability of the dynamics during the inter-outbreak period will scale with the size of the susceptible population.

Note heuristically in Eq. (2) that climatic variability could drive the parameter *β* and thereby influence *λ*. Indeed, for continuous seasonally recurring epidemics, outbreaks are often thought of as being driven by climatic conditions that vary between seasons,  with some of the year-to-year variability in outbreak magnitude attributed to multiyear climate variability^17,18^. Thus, it is reasonable to view changes in *λ* immediately leading to the transition as being driven by slow changes in *β* that reflect the pulse of the seasonal climatic drivers. Because one annual cycle consists of a stable inter-outbreak period (where *λ* < 0) followed by a critical transition as *λ* becomes positive, *λ* approaching 0 becomes an early-warning indication of the imminent outbreak^19^. Figure 1 shows a schematic illustration of this mixed modeling scheme using the SIR model in Eq. (1), with a seasonally varying (sinusoidal) *β* with no inter-annual variability, and randomly reset values of *S*.

The general mathematical argument that *λ* is a proxy for susceptibles is presented in the Methods. The scheme encompasses a wide range of mathematical models, from the simple SIR models to the most complex vector-host models. Supplementary Fig. 4 demonstrates the method’s robustness using an SIR model with a recruitment term that allows for stochastic jumps of random sizes, at random times.

### Dengue in San Juan, Puerto Rico

We demonstrate the approach with an analysis of the record of dengue hospitalizations in San Juan, Puerto Rico. To validate the mixed modeling framework asserted in Fig. 1, we perform an empirical dynamic analysis on the data (EDM)^20,21^ (see brief introductory animation http://tinyurl.com/EDM-intro^12^). Figure 2 shows that the inter-outbreak periods have an optimal embedding dimension, *E* = 3 and linear equilibrium dynamics consistent with local contraction of the dynamics onto a point equilibrium (random linear displacements around a fixed point), while the outbreaks involve regions of the attractor that are significantly higher dimensional, *E* = 9, and exhibit nonlinear dynamics (see Methods). This evidence supports the theoretical set up as described above.

**Fig. 3:**
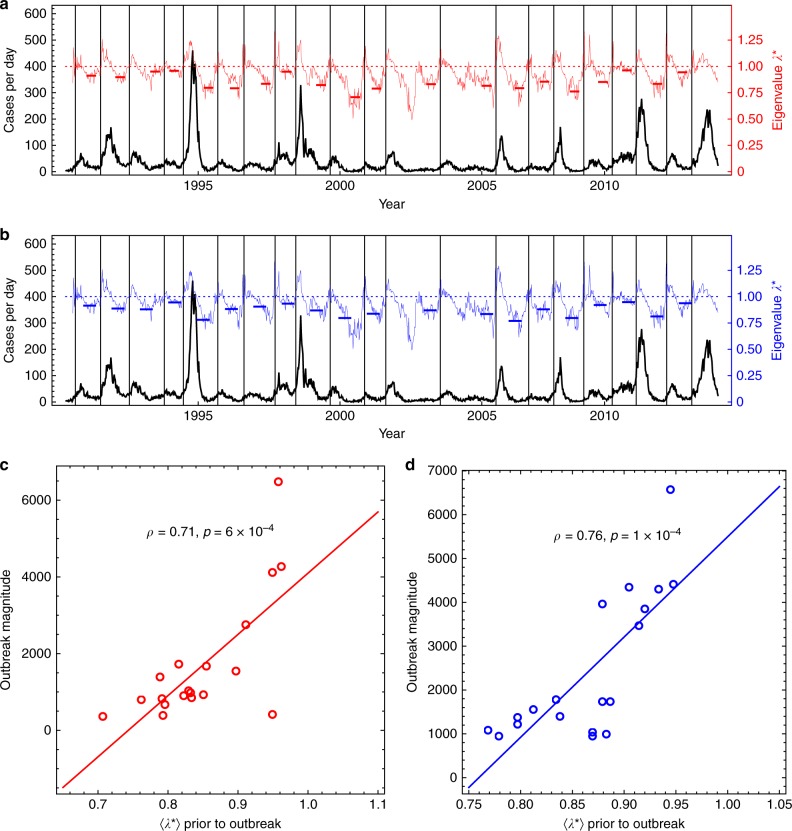
Prediction of dengue outbreak magnitudes in San Juan. **a** The back lines show the incidence time series and the thin red curve shows the time series for the eigenvalues $\lambda _t^ \ast$ calculated in 12-week running windows. The thick red horizontal bars represent the average value of the eigenvalue λ* in the assessment interval (the proxy for estimating susceptibles) calculated 12 weeks prior to the onset of an outbreak (as defined dynamically where $\lambda _t^ \ast \, > \, 1$ (see Methods)). **b** As in (**a**) with the proxy 〈λ*〉 calculated on an arbitrary fixed date 16 weeks prior to September 1 with an arbitrary 16-week assessment interval. A detailed analysis of robustness to the choice of assessment interval is given in Supplementary Figs. 1 and 2. By definition (*λ* > 0) there are no outbreaks in 2003 and 2005. **c** shows the correlation between predictors and the subsequent outbreak sizes using the onset protocol in (**a**). **d** As in (**c**), but for the fixed-time protocol in (**b**)

With discrete weekly incidence data *I*(*t*) the stability of the system during the inter-outbreak period is quantified by the discrete-time eigenvalue or “multiplier” λ* constructed as an average of local eigenvalues $\lambda _t^ \ast$. (Note that $\lambda = \frac{{{\mathrm{ln}}(\lambda ^ \ast )}}{{{\mathrm{\Delta }}t}}$ provides the leading eigenvalue of the linearized continuous dynamics.) These $\lambda _t^ \ast$ are calculated by linear regression of the relation *I*(*t* + Δ*t*) = λ*I(*t*) for values of *t* in a 12-week running window with Δ*t* = 1 week (see Methods for robustness to parameter choice). The resulting time series of local values of $\lambda _t^ \ast$ are shown by the thin red and blue lines in Fig. 3a, b. Clearly, during stable inter-disease periods λ* < 1, and outbreak onsets occur when λ* exceeds unity, thus λ* > 1 is an early-warning indicator.

The proxy for susceptibles λ* is calculated as an average of $\langle \lambda _t^ \ast \rangle$ over a time interval sufficiently long to reliably estimate $\langle \lambda _t^ \ast \rangle$ and which is far enough in advance to be useful. We denote the period over which values of $\lambda _t^ \ast$ are averaged the “assessment interval”. Thus, in Fig. 3a, c the proxies $\langle \lambda _t^ \ast \rangle$ are calculated with a 12-week assessment window ending 3 months prior to onset (here defined by λ* > 1) and beginning with data 6 months prior (Methods). Figure 3c shows the performance measured by the Pearson correlation between $\langle \lambda _t^ \ast \rangle$ and the observed outbreak magnitude. It is 0.71 (*p* = 6 × 10^−4^). Importantly, these results are robust to how the assessment interval is selected, how the magnitude of an outbreak is defined, and how the predictor is constructed (see Supplementary Figs. 1 and 2). This robustness is highlighted in Fig. 3b, d with an assessment interval that is arbitrarily fixed by calendar day. The correlation between the predictors $\langle \lambda _t^ \ast \rangle$ and the outbreak magnitudes is 0.76, with *p* = 5 × 10^−5^.

### A generic approach

**Fig. 4:**
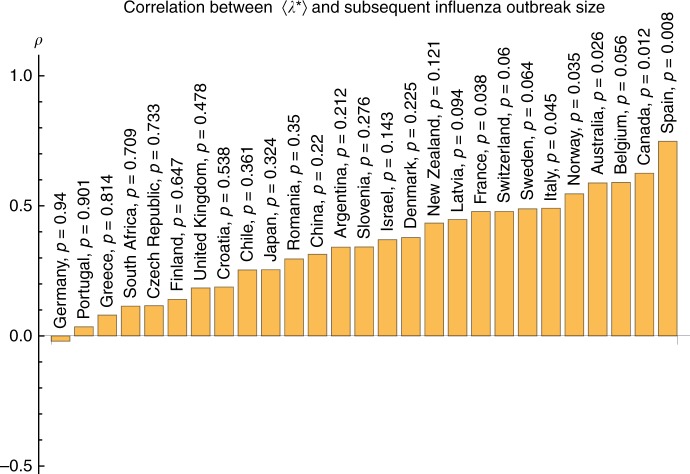
Demonstration of the method for global influenza. Shows the correlations between the eigenvalues 〈λ*〉 and the subsequent influenza outbreak magnitudes in the 27 different countries having less than 80% missing values during the assessment period. Details in the Methods (Supplementary Figs. 5–31 for each country)

This prediction scheme is conceived to be generic for seasonal epidemics, and its performance will vary with the specifics of the disease, including how reliable the inter-outbreak data are (affected by the severity of the disease or lack thereof), the potential role of climatic drivers during the outbreak (such as the massive hurricane in 2017) and particularly how soon before the new outbreak the new virus has evolved (whether there is sufficient advance time to assess λ*). To test its generality we examined records of influenza incidence gathered from the World Health Organization database, where we identified 27 countries with data having less than 80% missing values during the assessment periods (see Methods). The results summarized in Fig. 4 show positive correlations between the λ*-based predictors and outbreak magnitudes for 26 of the 27 countries. Supplementary Fig. 32 shows the corresponding analyses for data on influenza-like illness in New York City and in the mid-Atlantic Census division, with results that are consistent with the results of the country-level influenza data, and slightly better for New York City. The generally weaker predictive skill (defined as the Pearson correlation) in these data than for dengue in San Juan could be related to many factors including (1) differences in the severity of the diseases with possible under-reporting of influenza in the inter-disease periods; (2) the fact that the coarser country-level flu data are generally less suitable for detecting low-dimensional dynamics^22^; (3) fundamental differences in the biology of the disease, such as a faster rate of evolution for the flu virus. (4) We also note that if novel strains of the influenza virus are introduced too close to onset of an outbreak, the assessment interval will not accurately reflect the susceptible pool, and the method should not perform. This was likely the case for countries in the southern hemisphere with respect to the 2009 swine-flu pandemic, a novel virus first described at the beginning of the southern hemisphere outbreak in April of that year (too late for estimating susceptibles there). However, in the northern hemisphere, the novel swine-flu signal arising by contagion from the south produced a clear dynamic signal over the summer inter-outbreak months in the north that allowed a good sampling of the dynamics to estimate the susceptible populations. This produced a skillful forecast of subsequent very large winter outbreaks in northern countries. The case can be seen by comparing Supplementary Figs. 5, 6 and 9, with e.g. Supplementary Figs. 15 and 20.

## Discussion

The results for dengue presented here are consistent with previous studies that have  built prediction models based on incidence data alone^23^. These studies show statistically that the “momentum” of the early part of the outbreak (the incidence in the late inter-disease period) can be associated with outbreak magnitude. However, the result is purely phenomenological and may be driven by something more fundamental. Our alternative mechanistic interpretation is based on the mathematical argument that dynamic stability should march with susceptibles. Thus, in theory, a stability metric should provide a good indicator for the magnitude of the subsequent outbreak. Thus, although momentum may be correlated, a dynamic proxy for susceptibles may represent  a better mechanistic explanation. The bottom-line is shown in Supplementary Fig. 33, where an analysis parallel to what is presented in Fig. 3 is given, except that the predictor used is the average number of hospitalizations 〈*I*(*t*)〉 over the assessment periods, instead of the average multipliers $\langle \lambda _t^ \ast \rangle$. As expected, the two predictors 〈*I*(*t*)〉 and $\langle \lambda _t^ \ast \rangle$ are positively correlated, but as shown in Supplementary Fig. 33, the more mechanistic average multipliers $\langle \lambda _t^ \ast \rangle$ perform better than statistical momentum 〈*I*(*t*)〉 as a predictor of outbreak magnitude.

Finally, Supplementary Fig. 34 shows how the demonstrated correlations can be used to make out-of-sample predictions of outbreak size and outbreak peaks. The model is a simple linear regression using $\langle \lambda _t^ \ast \rangle$ as a predictor variable trained on the first six outbreaks of the time series of dengue hospitalizations in San Juan, and tested on the last 13 outbreaks.

## Methods

### The general framework for estimating susceptibles

Consider ordinary differential equations of the form (classical epidemic models (SIR and SIRS models) are special cases)

$$(\mathop {{\mathbf{I}}}\limits^. ,\mathop {{\mathbf{S}}}\limits^. ) = {\mathbf{F}}({\mathbf{I}},{\mathbf{S}};\beta ),$$

where **I** = (*I*_1_,…, *I*_*n*_) and **S** = (*S*_1_,…, *S*_*n*_) represent infected and susceptible population sizes (humans and/or vectors) respectively, *β* represents time-varying system parameters (e.g. seasonal climate variables), and **F** is a generally unknown nonlinear function. By disregarding birth and death rates, the condition **F**(0, **S**; β) = 0 for all **S** and *β* indicates that any disease-free state of the system (inter-outbreak period) represents an equilibrium that is independent of the values of **S** and *β*. Because the stability of an inter-outbreak period is determined by the Jacobian matrix *D***F**(0, **S**; *β*), and since **F** is constant in the plane **I** = 0, it holds that

$$\frac{{\partial {\mathbf{F}}}}{{\partial {\mathbf{S}}}}|_{{\mathbf{I}} = 0} = 0.$$

The latter shows that the Jacobian *A* is singular in the inter-outbreak period so that the linearized dynamics of **I** has reduced dimensionality: $\mathop {{\mathbf{I}}}\limits^. = A({\mathbf{S}}^ \ast ,\beta ){\mathbf{I}}$, where

$$A = \frac{{\partial {\mathbf{F}}}}{{\partial {\mathbf{I}}}}|_{{\mathbf{I}} = 0},$$

and S* is a parameter. This simplification means that the stability of the system is determined by *A*’s leading eigenvalue *λ*, which crucially depends on S* and *β*. The practical beauty of the dimensionality reduction is that the leading eigenvalue can be estimated from the incidence data by sampling the time series data of **I**, during the inter-outbreak periods, where linearization is a reasonable approximation. Thus, regardless of the complexity of the attractor during the outbreak itself, or subsequently where the dynamics are so high dimensional as to be effectively stochastic, the leading eigenvalue during the subsequent stable inter-outbreak period should be a proxy for the realized value of S* determining the outbreak. Again, this general framework encompasses a wide range of mathematical models, from the simple SIR models to the most complex vector-host models (see below).

### A note on *λ* in the Bailey−Dietz model

The classical Bailey−Dietz model for vector-transmitted diseases model has the form

$$\begin{array}{l}\dot S_h = \Lambda _h - \beta _1S_hI_v - \mu _hS_h\\ \dot I_h = \beta _1S_hI_v - \gamma _hI_h - \mu _hI_h\\ \dot R_h = \gamma _hI_h - \mu _hR_h\\ \dot S_v = \Lambda _v - \beta _2S_vI_h - \mu _vS_v\\ \dot I_v = \beta _2S_vI_h - \mu _vI_v\end{array}.$$

By omitting the deterministic recruitment rates and death rates, the system becomes

$$\begin{array}{l}\dot S_h = - \beta _1S_hI_v\\ \dot I_h = \beta _1S_hI_v - \gamma _hI_h\\ \dot R_h = \gamma _hI_h\\ \dot S_v = - \beta _2S_vI_h\\ \dot I_v = \beta _2S_vI_h\end{array}.$$

for which any disease-free state ($S_h = S_h^ \ast$, *I*_*h*_ = 0, $S_v = S_v^ \ast$, *I*_*v*_ = 0) is an equilibrium. At a disease-free equilibrium, the Jacobian has rank 2, and the linearlized equations for *I*_*h*_ and *I*_*v*_ are

$$\begin{array}{l}\dot I_h = \beta _1S_h^ \ast I_v - \gamma _hI_h\\ \dot I_v = \beta _2S_v^ \ast I_h\end{array}$$

and the eigenvalues are

$$\lambda = \frac{{\gamma _h}}{2} \pm \sqrt {\left( {\frac{{\gamma _h}}{2}} \right)^2 + \beta _1\beta _2S_h^ \ast S_v^ \ast } .$$

In particular, the leading eigenvalue is an increasing function of the susceptible populations $S_h^ \ast$ and $S_v^ \ast$.

### Empirical dynamic modeling study (EDM)

Empirical dynamic modeling involves studying system dynamics from an attractor (or in our case, attractor regions) constructed from time series (see brief introductory animation http://tinyurl.com/EDM-intro; SI of ref. ^12^). If system behavior is governed by deterministic rules, then attractor manifolds exist, and these can be built from lags of a single variable^21^, or multivariately from combinations of variables^13,24,25^. The details and code for EDM including a tutorial describing the specific analyses undertaken here for computing the optimal embedding dimension (Fig. 2a) and the S-map test for nonlinearity (Fig. 2b) are found in refs. ^12,21,24^ and on CRAN for rEDM https://cran.r-project.org/web/packages/rEDM/vignettes/rEDM_tutorial.html

### Estimation of ${\mathbf{\lambda }}_t^ \ast$ and definition of outbreak onset

From time series data *I*(*t*) of dengue incidence in San Juan, we construct a time series of discrete-time eigenvalues (local multipliers) $\lambda _t^ \ast$. Thus, we identify segments or windows

$$W_t = (I(t - T),I(t - T + 1), \ldots ,I(t))$$

of length *T* = 12 weeks and estimate a local discrete eigenvalue or multiplier, denoted $\lambda _t^ \ast$ by performing a linear regression (with zero intercept) of the model *I*(*t*′ + 1) = λ^*^I(*t*′) using the data contained in each window *W*_*t*_. Thus, the time series of $\lambda _t^ \ast$ is generated from the moving 12-week windows, where each discrete eigenvalue $\lambda _t^ \ast$ depends only on the values of *I* in each window *W*_*t*_. Note that $\lambda _t^ \ast$ is computed from a locally varying Jacobian but is not a local Lyapunov exponent as for S-maps^21^. To define the onset of a disease outbreak dynamically, we construct a smoothed version $\tilde \lambda _t^ \ast$ of the signal $\lambda _t^ \ast$ using an arbitrary10-week moving average. Supplementary Fig. 1 shows that results are robust to the specific length of the moving average. A time *t*_*c*_ is called an outbreak onset if $\tilde \lambda _{t_c - 1}^ \ast \, < \, 1$ and $\tilde \lambda _{t_c}^ \ast \, > \, 1$. We note that because dengue in San Jua\n is predictably seasonal, the dynamic definition of onset coincides closely with a fixed calendar date for onset (September 1) allowing for better advance warning.

### Assessment intervals and robustness

To predict outbreak magnitude, we compute an average multiplier (average discrete-time eigenvalue) over a period of time that is far enough in advance of the outbreak (so as to be useful and not to include the outbreak dynamics), but which samples the final inter-outbreak dynamics sufficiently to reliably estimate an effective eigenvalue proxy for the susceptibles. We denote this period over which values of $\lambda _t^ \ast$ are averaged by $\tilde J$, and denote the assessment interval *J* as the segment of the incidence time series which is used to obtain $\langle \lambda _t^ \ast \rangle$. The assessment interval *J* includes the *T* − 1 weeks prior to the beginning of $\tilde J$ since $\lambda _t^ \ast$ are obtained in running windows of length *T*. The assessment interval is defined by its advance prediction time *t*_1_ (the time between the end of the assessment interval and the onset of the subsequent outbreak), and its length *n*, i.e.

$$\tilde J = \{ t_c - t_{n - T + 1}, \ldots ,t_c - t_2,t_c - t_1\} ,$$

with *t*_*k*+1_ = *t*_*k*_ + (1 week), and

$$J = \{ t_c - t_n, \ldots ,t_c - t_2,t_c - t_1\} .$$

Thus, the susceptible proxy 〈λ^*^〉 is computed as the average *μ*_*t*_ in *J*:

$$\langle \lambda ^ \ast \rangle = \frac{1}{n}\mathop {\sum}\limits_{t \in \tilde J} {\lambda _t^ \ast } .$$

For the analysis presented in Fig. 2a, c, the values *t*_1_ = 12 weeks, and *n* = 24 weeks were used; however, the results are robust to the choice of *t*_1_ and *n* (see Supplementary Fig. 1). The average multipliers 〈λ*〉 are compared with the subsequent outbreak magnitudes, yielding a correlation coefficient *ρ* = 0.71 with *p* = 6 × 10^−4^. The outbreak magnitude here is defined as the total number of reported cases within a year in the time period from outbreak onset to the beginning of the subsequent assessment interval. The robustness of the results was tested by computing the correlation coefficient *ρ* and the *p* value for a range of *t*_1_ values and *n* values. The results of this robustness analysis are presented in Supplementary Figs. 1 and 2. To simplify the robustness analysis, the outbreak magnitudes are defined as the total number of reported cases in the year following the outbreak onset.

In Supplementary Fig. 3a, c we present an analysis similar to Fig. 2a, c except that here we are estimating the peak of the outbreak (defined as the maximum number of reported cases in a week in the time period between the outbreak onset and the beginning of the subsequent assessment period). The corresponding robustness analysis is presented in Supplementary Fig. 4.

### Analysis of influenza data

The influenza analysis used time windows of length *T* = 30 weeks to calculate *μ*_*t*_ and an assessment interval of 5 weeks (includes incidence values beginning 35 weeks prior to an outbreak). This means that the proxy calculation is made 5 weeks prior to the outbreak. Outbreak onsets were defined dynamically as described above, and the outbreak magnitudes were defined as the total number of reported cases per capita in the year following the outbreak onsets.

Although the influenza database includes 79 different countries, only about half of these had sufficient data (free of excessive missing values) for a meaningful analysis. For a country to be included in the analysis, we liberally required at least ten assessment periods, with subsequent outbreak periods that each contain data for at least 20% of its weeks. The time series from 27 countries meet this minimal condition, and the prediction results for these countries are shown in Fig. 4.

### Construction of Fig. 2

Figure 2 justifies the mixed modeling approach with an empirical dynamical modeling (EDM) study on the outbreak periods and the inter-disease periods of the time series of dengue incidence in San Juan. Full details and code for such a study are found on CRAN for rEDM (https://rdrr.io/cran/rEDM/).

Figure 2a shows how the predictive skill (Pearson correlation) with simplex projection varies with embedding dimension. The inter-disease periods are defined as the time series segments containing data points between 30 and 12 weeks prior to the outbreak onsets. This yields 20 time series segments. The simplex predictions were made out of sample by using the first ten of these to construct the “library” attractor that is used to forecast the last ten segments out of sample.

Briefly, simplex projection is forecasting using nearest neighbor analogs to the *E*-dimensional vectors

$$v_t = (I(t - E + 1), \ldots ,I(t - 1),I(t)).$$

To each of the vectors *v*_*t*_ we associate the value *I*(*t* + 1), requiring that *I*(*t* + 1) belongs to the same segment as *I*(*t*). The association $v_t \, \mapsto \, I(t + 1)$ defines a real-valued function in *E* variables, that we denote by *F*. *F* constructed from the first ten segments is used to make out-of-sample prediction on the test data consisting of the last ten segments of the time series. We reconstruct attractors in each segment by taking vectors

$$w_t = (I(t - E + 1), \ldots ,I(t - 1),I(t))$$

and compute the 2-norm distances

$$d_{ts} = \left\| {w_t - v_s} \right\|,$$

for each *v*_*s*_ in the library. The prediction of *I*(*t* + 1) is the weighted average

$$I_{{\mathrm{pred}}}(t + 1) = \frac{1}{M}\mathop {\sum}\limits_{i = 1}^{E + 1} {e^{ - d_{ts(i)}}} F(s(i)),$$

where *d*_*ts*(1)_ ≤ *d*_*ts*(2)_ ≤ ⋯ ≤ *d*_*ts*(*E*+1)_ are the *E* + 1 smallest distances between library vectors and the vector *w*_*t*_, and *M* is a normalizing factor. The red curve in Fig. 2a shows the correlation between *I*(*t* + 1) − *I*(*t*) and the predicted values *I*_pred_(*t* + 1) − *I*(*t*) as functions of the embedding dimension *E*. The red curve shows a low optimal embedding dimension *E* = 3 consistent with collapse to stable states and the blue curve shows the same analysis for the outbreak periods (defined as the 30 weeks following disease onsets) yields a higher optimal *E* = 9.

Figure 2b shows the S-map test for nonlinearity^20^ and shows the predictive skill (Pearson correlation) of the S-map predictors as functions of the localization parameter *θ*. If predictability increases for values of *θ* > 0, then nonlinear state dependence is established (localization on the attractor is important). For the inter-disease periods an embedding dimension *E* = 3 is used and for the outbreaks *E* = 9 is used. S-map predictions involve constructing linear models for each point predicted using weighted multivariable linear regression

$$I(t + 1) = \alpha _0 + \alpha _1I(t - E + 1) + \cdots + \alpha _EI(t)$$

That is, points on the library attractor are given exponential weight depending on how close on the attractor they are to the predictee, and this is used for computing the Jacobian coefficients by SVD. That is, a linear surface is fit to each point on the attractor, meaning that for each test vector predicted a linear model is computed by weighing the data points *y* = *I*(*s* + 1) and *x* = *v*_*s*_ in the library by a factor proportional to $e^{ - \theta d_{ts}/\langle d\rangle }$ where 〈*d*〉 is the mean (2-norm) distance between the predictee and the library vectors *v*_*s*_. Hence, the estimated regression parameters $\hat \alpha _k$ (the sequentially computed Jacobian coefficients determined by SVD) depend on via the predictee on the localization parameter *θ*. Thus, the predicted value

$$I_{{\mathrm{pred}}}(t + 1) = \hat \alpha _0 + \hat \alpha _1I(t - E + 1) + \cdots + \hat \alpha _EI(t)$$

depends on the nonlinear control parameter *θ*. Figure 2b shows how the predictive skill of the S-map method varies with this parameter. If prediction skill increases for any value of *θ* > 0, nonlinear state dependence is established which indicates nonequilibrium nonlinear dynamics during outbreaks (blue curve). If not, the dynamics are essentially linear-stochastic and stable but driven by external perturbation (red curve).

### Reporting summary

Further information on research design is available in the Nature Research Reporting Summary linked to this article.

## Supplementary information

Supplementary Information

Reporting Summary

## Data Availability

Data of dengue incidence are from http://dengueforecasting.noaa.gov. The data of influenza incidence are from http://www.pnas.org/content/suppl/2016/10/26/1607747113.DCSupplemental/pnas.1607747113.sd01.txt. Only studies with fewer than 80% of values missing were included. All other data are available from the authors upon reasonable request.

## Code availability

The code used to analyze data is available from the first author upon request. General code for EDM analysis is available on CRAN at https://rdrr.io/cran/rEDM/.
