# Supplementary material for: Inter-outbreak stability reflects the size of the susceptible pool and forecasts magnitudes of seasonal epidemics
Source: Nat Commun. 2019 May 30;10:2374. doi: 10.1038/s41467-019-10099-y (PMC6542824; doi:10.1038/s41467-019-10099-y)
Supplement: Supplementary file 1 — Supplementary Information [file 41467_2019_10099_MOESM1_ESM.pdf]

Supplementary information

Inter-outbreak stability reflects the size of the susceptible pool and forecasts magnitudes of seasonal epidemics

Martin Rypdal[1] and George Sugihara[2,*]

[1] *Department of Mathematics and Statistics, UiT The Arctic University of Norway, Norway.*

[2] *Scripps Institution of Oceanography, University of California San Diego, La Jolla, CA, USA.*

Supplementary Figures

Supplementary Figure 1: Robustness of predictive skill for dengue in San Juan.

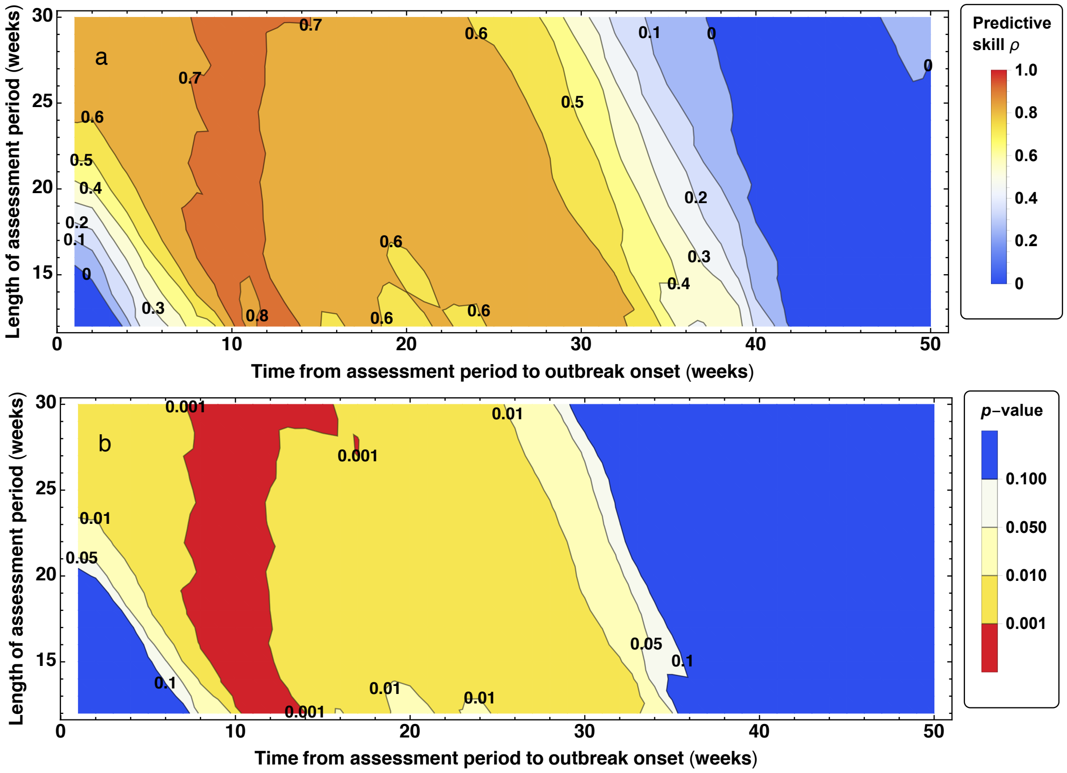

a, Shows the correlation ρ (the predictive skill) between outbreak magnitude and the estimated multiplier averaged using 1500 different definitions of the assessment intervals. Each definition of the assessment intervals is determined by its length (in weeks) and the time duration from the end of the assessment period to the outbreak onset. **b**, The p-values associated with the correlations in a.

Supplementary Figure 2: Prediction of dengue outbreak peak magnitudes in San Juan.

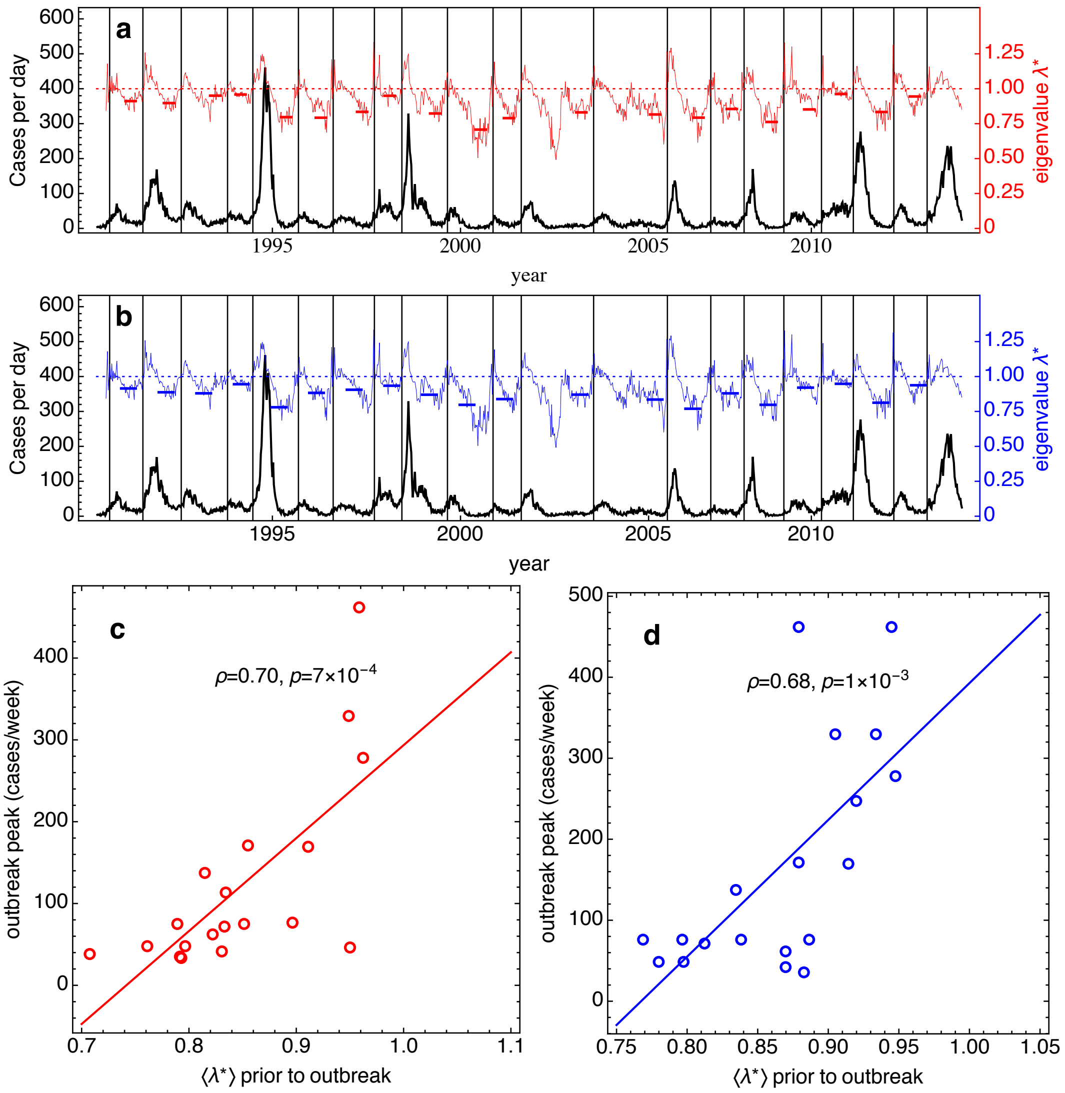

Shows the same analysis as in Fig. 3, but where the outbreak peak magnitude is predicted (defined as the maximal weekly number of cases during the outbreak period), rather than the cumulative magnitude of the outbreak. As in Fig. 3 results using a dynamically defined out break ($\lambda_t^* > 1$) are in red, while those using a fixed date are shown in blue. **a**, Same as Fig. 3a. **b**, Same as Fig. 3b. **c**, Shows the correlation between predictors and the subsequent outbreak peaks using the onset protocol in a. **d**, As in c, but for the fixed-time protocol in b.

Supplementary Figure 3: Robustness of predictive skill for peak outbreak magnitude dengue in San Juan.

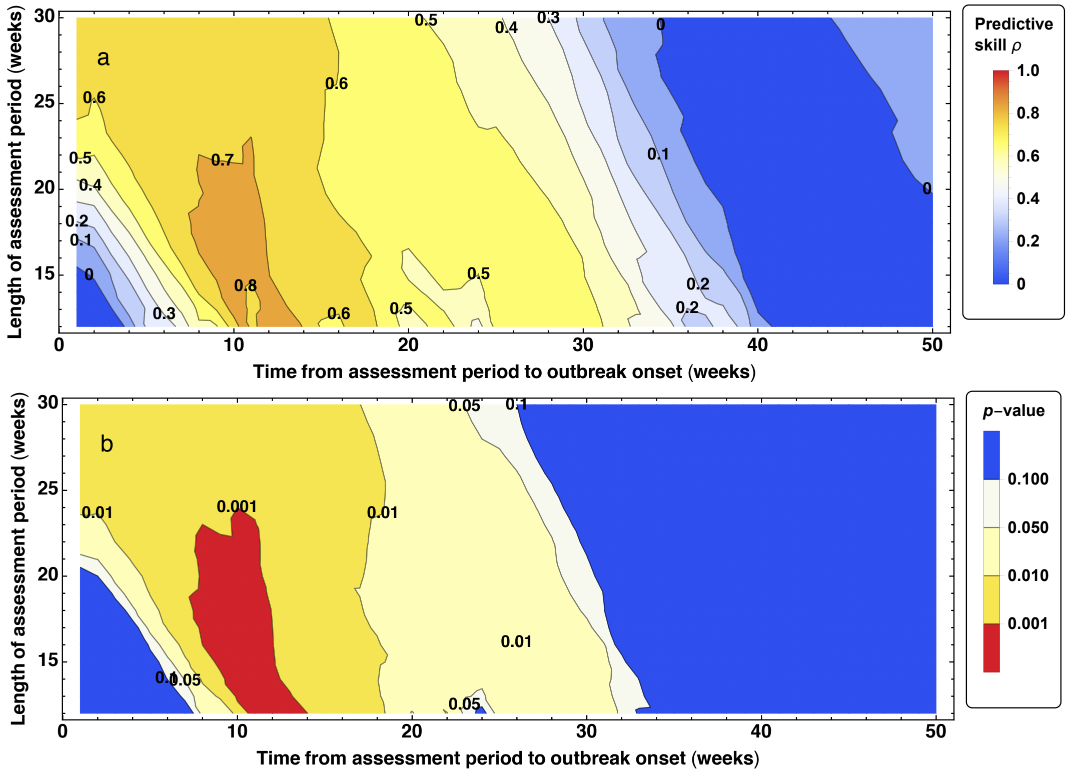

Shows the same analysis as in Extended Data Fig. 1, but where the peaks of the outbreaks (defined as the maximal weekly number of cases during the outbreak period) is predicted, rather than the outbreak magnitude. **a**, The correlation ρ (the predictive skill) between outbreak peaks and the estimated multiplier averaged using different definitions of the assessment intervals. **b**, The p-values associated with the correlations in a.

Supplementary Figure 4: Analysis of SIR model realizations.

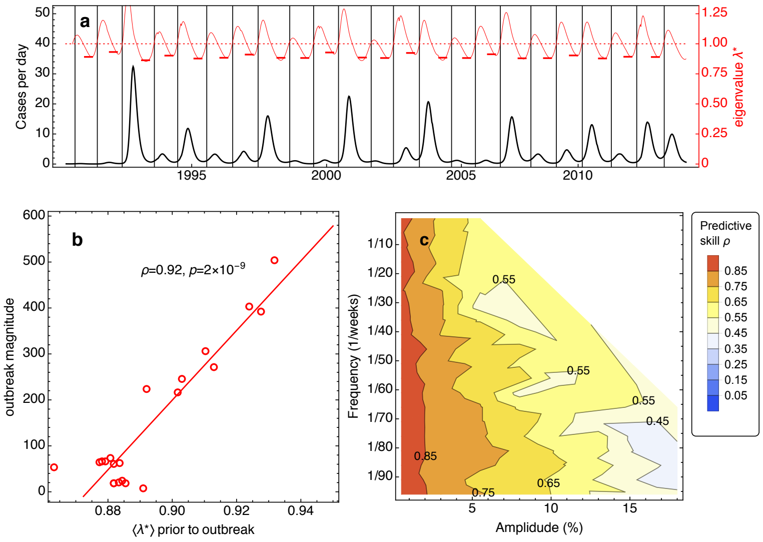

The simulated model is $\dot{I} = \beta(t)IS - \gamma I$ and $\dot{S} = -\beta(t)IS + \dot{\mu}$ with $\beta(t) = a + b(1 - \cos(2\pi f t))$ and $\mu(t) = \mu_0 t + J(t)$, where $J(t)$ is a Poisson jump process. In the jump process, the time between jumps and the jump sizes are drawn from exponential distributions, and the deterministic recruitment rate μ_0 is chosen so that the cumulative recruitment over the length of the time series is independent of the two exponential distributions that characterize the process $J(t)$. In all simulations, the parameter values are $a = 0.00005$, $b = 0.0085$, and $f = 1/(52 \text{ weeks})$. **a**, Shows an example where the mean time between jumps is 66 weeks and the mean jump size is 5% of the population in the model. **b**, For the example in a, this shows the predictive skill of using the average eigenvalue over assessment periods in the inter-disease periods. **c**, Evaluation of the method's predictive skill for varying parameters in the jump process $J(t)$. An ensemble of 100 realizations are run for each parameter value, and the presented correlations are the averages of these. The axis for amplitude represents the mean size of the jumps in the stochastic recruitment process, and the frequency-axis refers to the mean number of jumps per week.

Supplementary Figure 5: Analysis of influenza in Argentina.

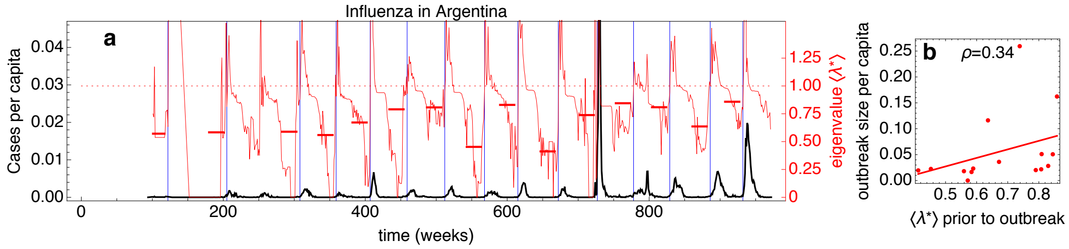

a, Influenza incidence (black) and the eigenvalue λ_t^* (red) for Argentina. **b**, Correlation between influenza outbreak magnitude and the eigenvalue estimated in the assessment interval.

Extended Data Figure 6: Analysis of influenza in Australia.

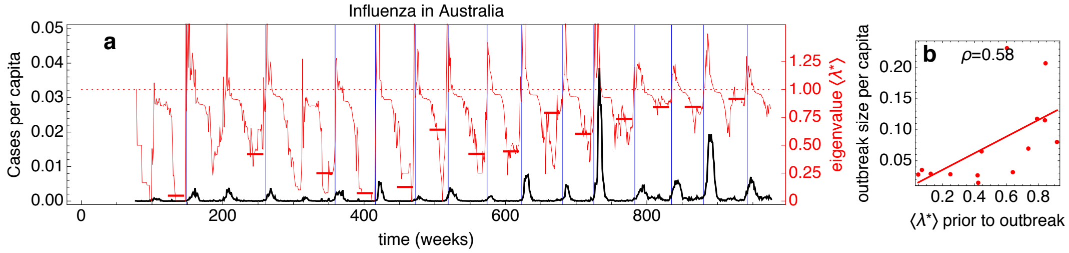

a, Influenza incidence (black) and the eigenvalue λ_t^* (red) for Australia. **b**, Correlation between influenza outbreak magnitude and the eigenvalue estimated in the assessment interval.

Supplementary Figure 7: Analysis of influenza in Belgium.

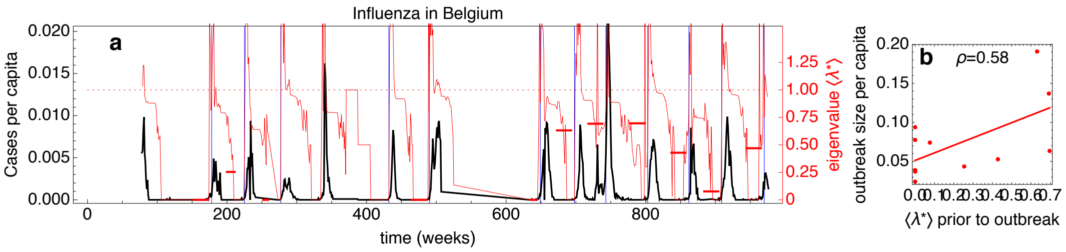

a, Influenza incidence (black) and the eigenvalue λ_t^* (red) for Belgium. **b**, Correlation between influenza outbreak magnitude and the eigenvalue estimated in the assessment interval.

Supplementary Figure 8: Analysis of influenza in Canada.

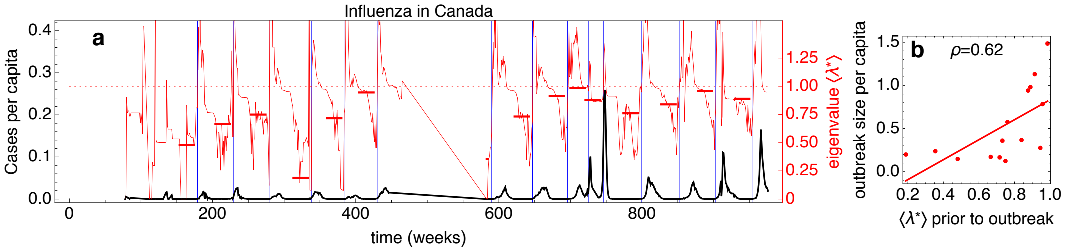

a, Influenza incidence (black) and the eigenvalue λ_t^* (red) for Canada. **b**, Correlation between influenza outbreak magnitude and the eigenvalue estimated in the assessment interval.

Supplementary Figure 9: Analysis of influenza in Chile.

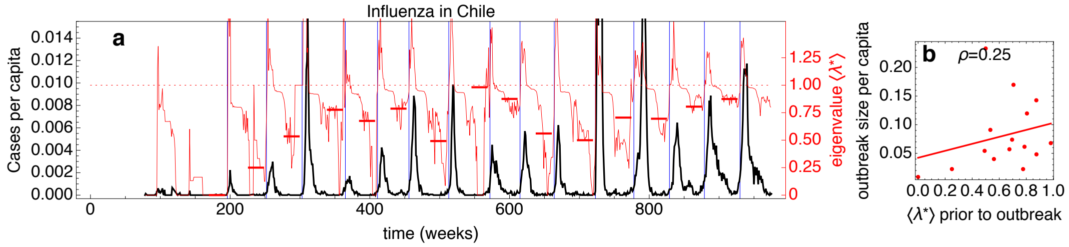

a, Influenza incidence (black) and the eigenvalue λ_t^* (red) for Chile. **b**, Correlation between influenza outbreak magnitude and the eigenvalue estimated in the assessment interval.

Supplementary Figure 10: Analysis of influenza in China.

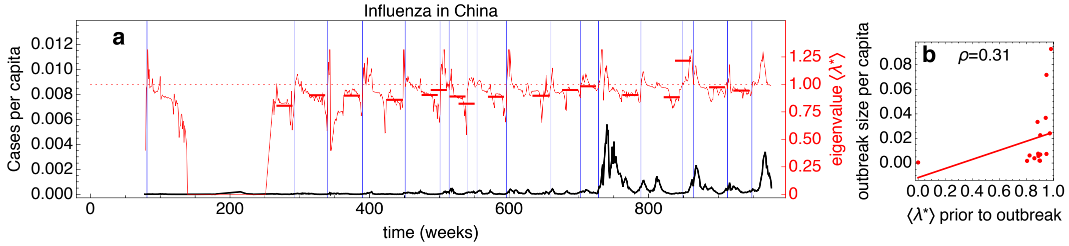

a, Influenza incidence (black) and the eigenvalue λ_t^* (red) for China. **b**, Correlation between influenza outbreak magnitude and the eigenvalue estimated in the assessment interval.

Supplementary Figure 11: Analysis of influenza in Croatia.

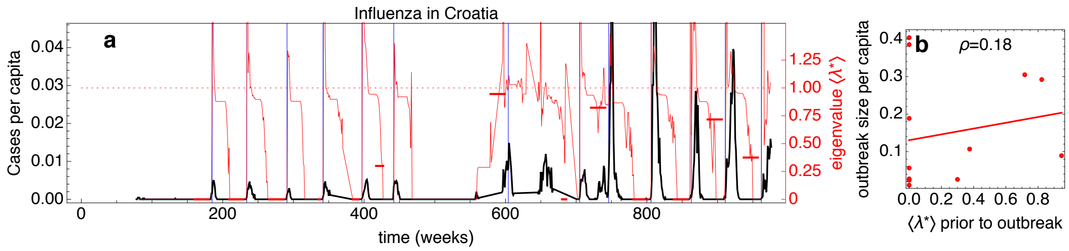

a, Influenza incidence (black) and the eigenvalue λ_t^* (red) for Croatia. **b**, Correlation between influenza outbreak magnitude and the eigenvalue estimated in the assessment interval.

Supplementary Figure 12: Analysis of influenza in Czech Republic.

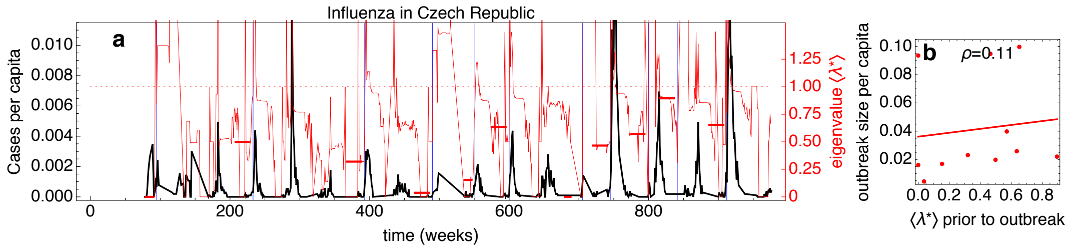

a, Influenza incidence (black) and the eigenvalue λ_t^* (red) for Czech Republic. **b**, Correlation between influenza outbreak magnitude and the eigenvalue estimated in the assessment interval.

Supplementary Figure 13: Analysis of influenza in Denmark.

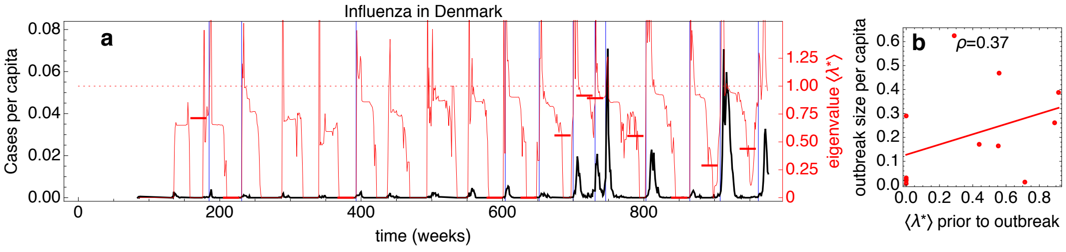

a, Influenza incidence (black) and the eigenvalue λ_t^* (red) for Denmark. **b**, Correlation between influenza outbreak magnitude and the eigenvalue estimated in the assessment interval.

Supplementary Figure 14: Analysis of influenza in Finland.

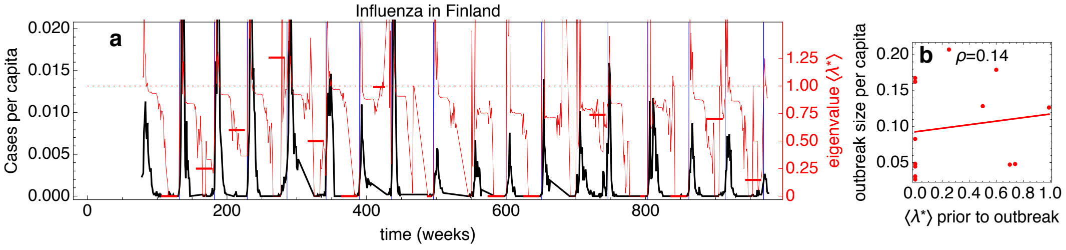

a, Influenza incidence (black) and the eigenvalue λ_t^* (red) for Finland. **b**, Correlation between influenza outbreak magnitude and the eigenvalue estimated in the assessment interval.

Supplementary Figure 15: Analysis of influenza in France.

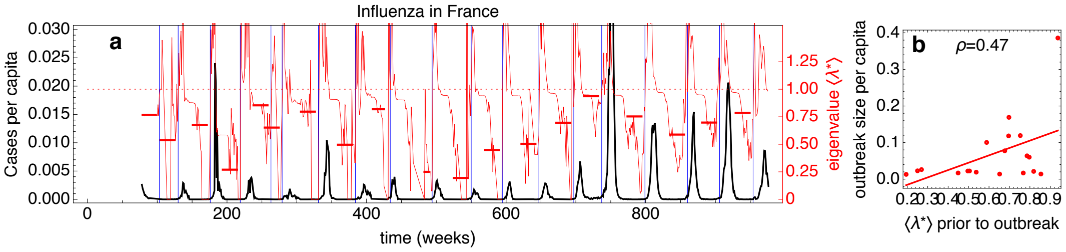

a, Influenza incidence (black) and the eigenvalue λ_t^* (red) for France. **b**, Correlation between influenza outbreak magnitude and the eigenvalue estimated in the assessment interval.

Supplementary Figure 16: Analysis of influenza in Germany.

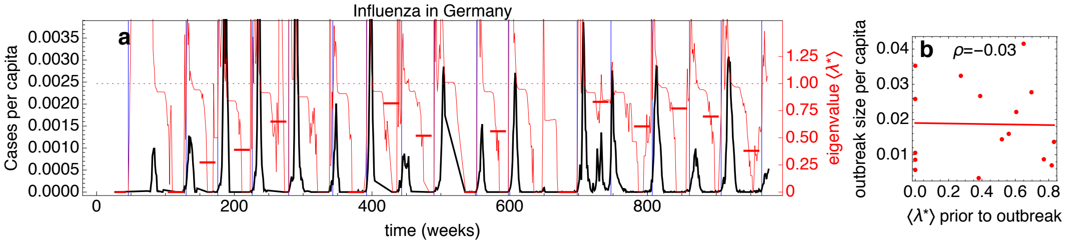

a, Influenza incidence (black) and the eigenvalue λ_t^* (red) for Germany. **b**, Correlation between influenza outbreak magnitude and the eigenvalue estimated in the assessment interval.

Supplementary Figure 17: Analysis of influenza in Greece.

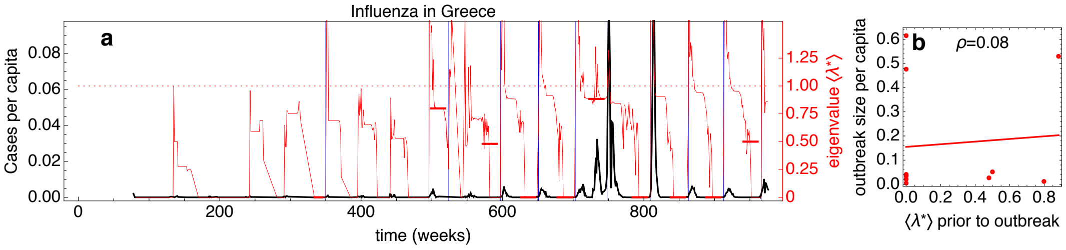

a, Influenza incidence (black) and the eigenvalue λ_t^* (red) for Greece. **b**, Correlation between influenza outbreak magnitude and the eigenvalue estimated in the assessment interval.

Supplementary Figure 18: Analysis of influenza in Israel.

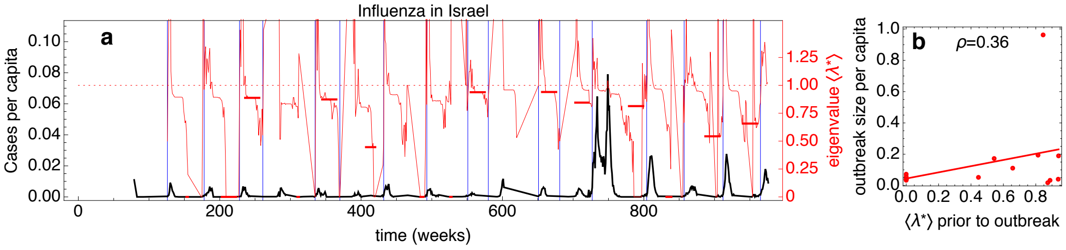

a, Influenza incidence (black) and the eigenvalue λ_t^* (red) for Israel. **b**, Correlation between influenza outbreak magnitude and the eigenvalue estimated in the assessment interval.

Supplementary Figure 19: Analysis of influenza in Italy.

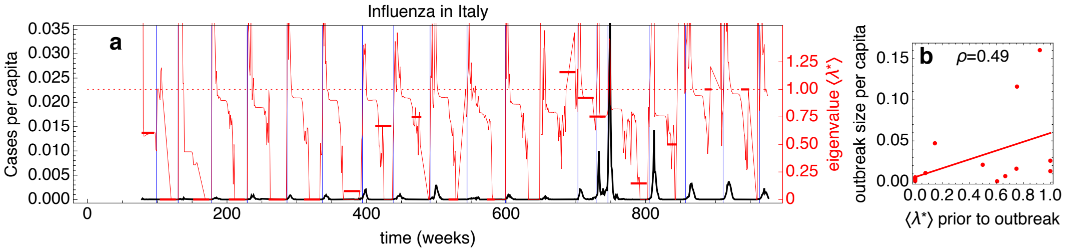

a, Influenza incidence (black) and the eigenvalue λ_t^* (red) for Italy. **b**, Correlation between influenza outbreak magnitude and the eigenvalue estimated in the assessment interval.

Supplementary Figure 20: Analysis of influenza in Japan.

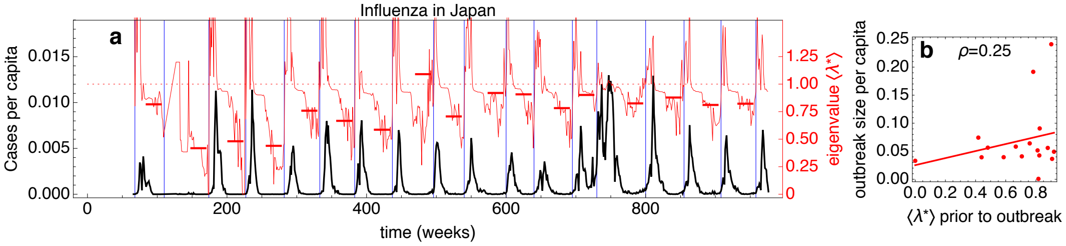

a, Influenza incidence (black) and the eigenvalue λ_t^* (red) for Japan. **b**, Correlation between influenza outbreak magnitude and the eigenvalue estimated in the assessment interval.

Supplementary Figure 21: Analysis of influenza in Latvia.

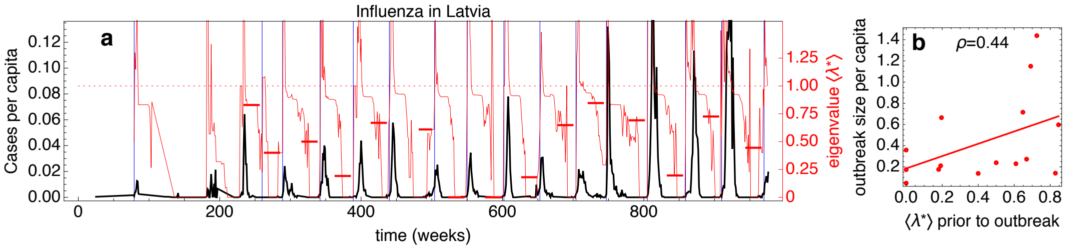

a, Influenza incidence (black) and the eigenvalue λ_t^* (red) for Latvia. **b**, Correlation between influenza outbreak magnitude and the eigenvalue estimated in the assessment interval.

Supplementary Figure 22: Analysis of influenza in New Zealand.

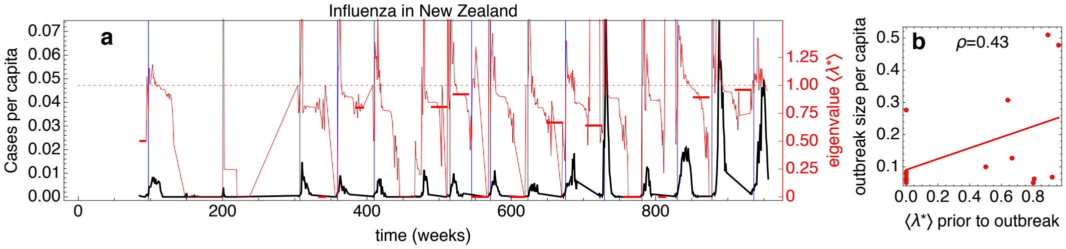

a, Influenza incidence (black) and the eigenvalue λ_t^* (red) for New Zealand. **b**, Correlation between influenza outbreak magnitude and the eigenvalue estimated in the assessment interval.

Supplementary Figure 23: Analysis of influenza in Norway.

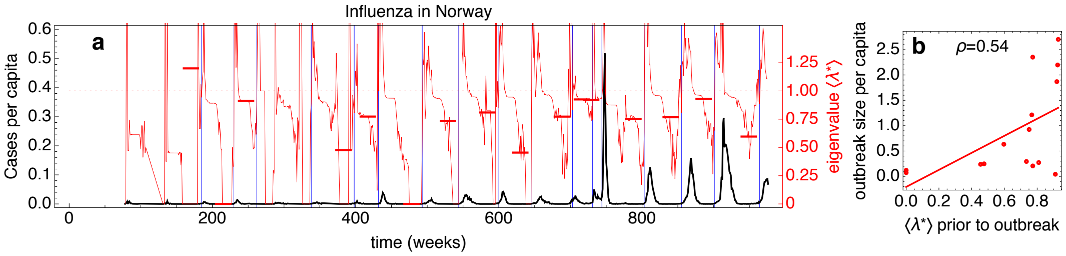

a, Influenza incidence (black) and the eigenvalue λ_t^* (red) for Norway. **b**, Correlation between influenza outbreak magnitude and the eigenvalue estimated in the assessment interval.

Supplementary Figure 24: Analysis of influenza in Portugal.

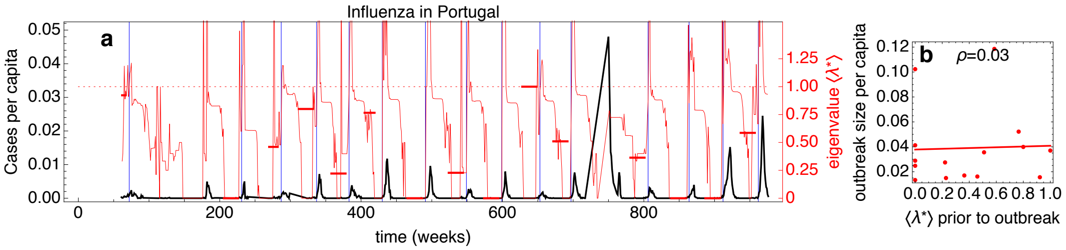

a, Influenza incidence (black) and the eigenvalue λ_t^* (red) for Portugal. **b**, Correlation between influenza outbreak magnitude and the eigenvalue estimated in the assessment interval.

Supplementary Figure 25: Analysis of influenza in Romania.

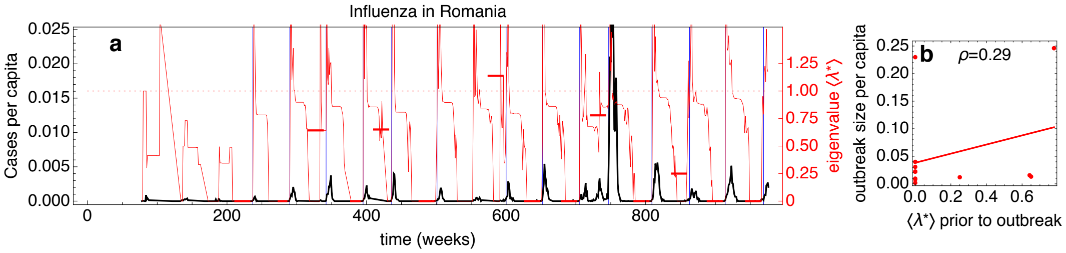

a, Influenza incidence (black) and the eigenvalue λ_t^* (red) for Romania. **b**, Correlation between influenza outbreak magnitude and the eigenvalue estimated in the assessment interval.

Supplementary Figure 26: Analysis of influenza in Slovenia.

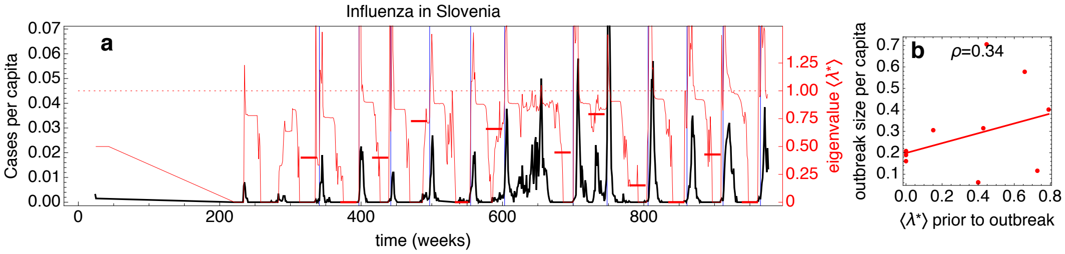

a, Influenza incidence (black) and the eigenvalue λ_t^* (red) for Slovenia. **b**, Correlation between influenza outbreak magnitude and the eigenvalue estimated in the assessment interval.

Supplementary Figure 27: Analysis of influenza in South Africa.

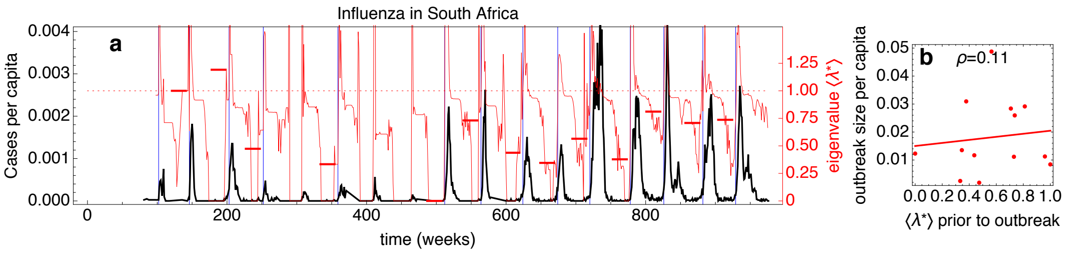

a, Influenza incidence (black) and the eigenvalue λ_t^* (red) for South Africa. **b**, Correlation between influenza outbreak magnitude and the eigenvalue estimated in the assessment interval.

Supplementary Figure 28: Analysis of influenza in Spain.

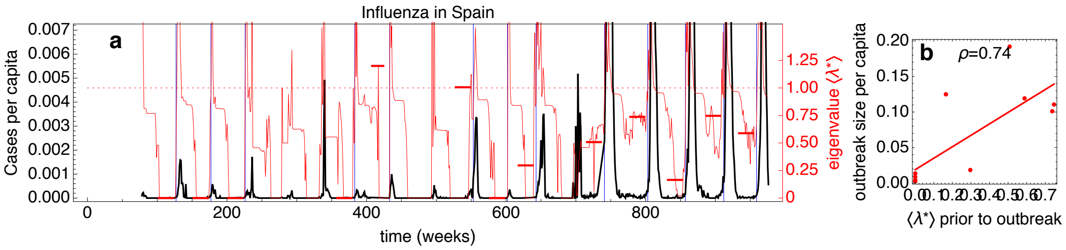

a, Influenza incidence (black) and the eigenvalue λ_t^* (red) for Spain. **b**, Correlation between influenza outbreak magnitude and the eigenvalue estimated in the assessment interval.

Supplementary Figure 29: Analysis of influenza in Sweden.

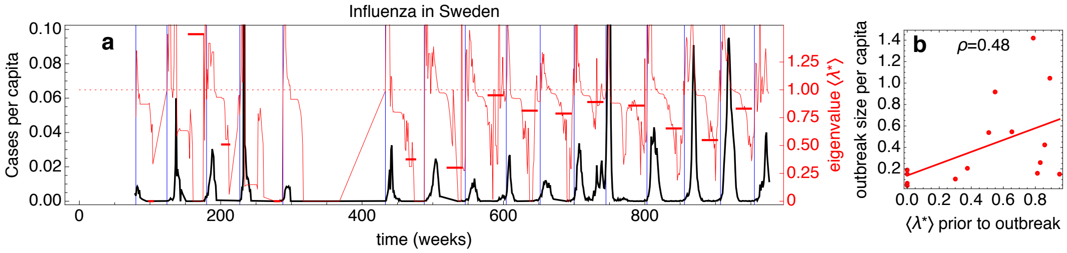

a, Influenza incidence (black) and the eigenvalue λ_t^* (red) for Sweden. **b**, Correlation between influenza outbreak magnitude and the eigenvalue estimated in the assessment interval.

Supplementary Figure 30: Analysis of influenza in Switzerland.

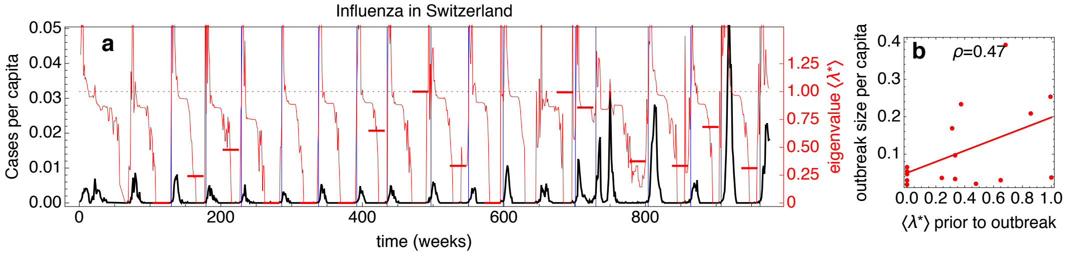

a, Influenza incidence (black) and the eigenvalue λ_t^* (red) for Switzerland. **b**, Correlation between influenza outbreak magnitude and the eigenvalue estimated in the assessment interval.

Supplementary Figure 31: Analysis of influenza in United Kingdom.

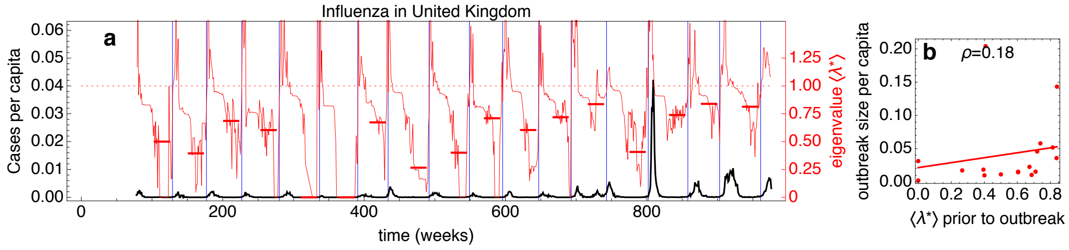

a, Influenza incidence (black) and the eigenvalue λ_t^* (red) for United Kingdom. **b**, Correlation between influenza outbreak magnitude and the eigenvalue estimated in the assessment interval.

156 **Supplementary Figure 32: Analyses of influenza-like illness in New York City and the mid-Atlantic Cencus division**

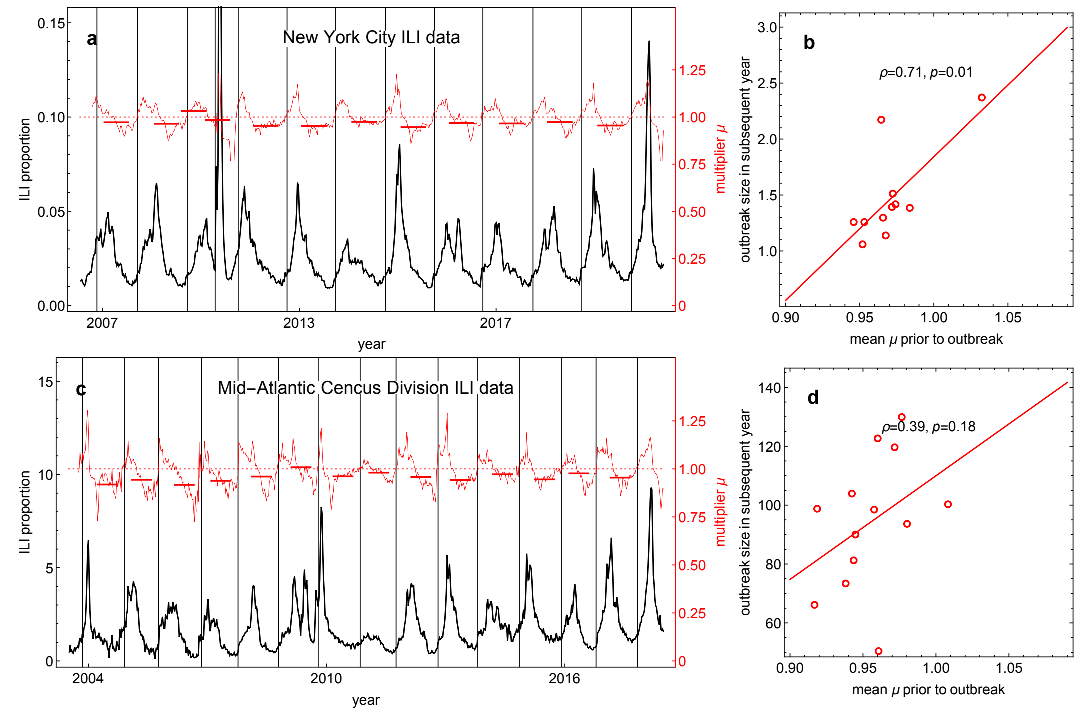

a, Incidence of influenza-like illness (black) and the eigenvalue λ_t^* (red) for New York City. **b**, Correlation between influenza outbreak magnitude and the eigenvalue estimated in the assessment interval for New York City. **c**, Incidence of influenza-like illness (black) and the eigenvalue λ_t^* (red) for the Mid-Atlantic Cencus Division. **d**, Correlation between influenza outbreak magnitude and the eigenvalue estimated in the assessment interval for Mid-Atlantic Cencus Division.

Supplementary Figure 33: Prediction of dengue outbreak magnitudes in San Juan using incidence in inter-disease periods.

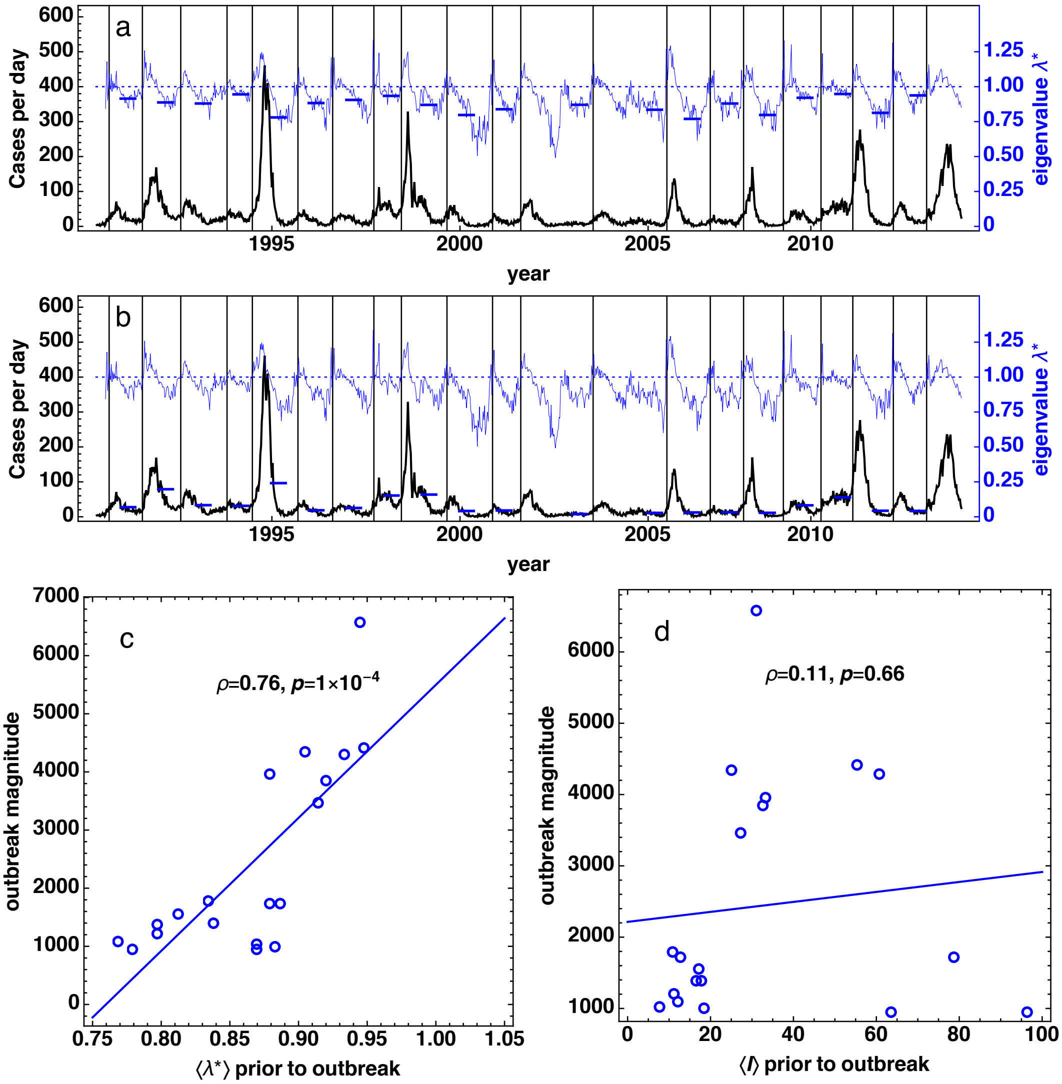

The figure shows a comparison of the analysis shown in Fig. 3b and 3d with the corresponding analysis using mean incidence as the predictor variable. **a**, Shows the same as Fig. 3b. **b**, As a, but the horizontal lines show the average incidence $\langle I \rangle$ instead of the the eigenvalue proxy $\langle \lambda^* \rangle$. **c**, Shows the same as Fig. 3d. **d**, As c, but but the eigenvalue proxy $\langle \lambda^* \rangle$ is replaced with the mean incidence $\langle I \rangle$ as the predictor variable.

Supplementary Figure 34: Trained prediction of outbreak magnitudes and outbreak peaks for dengue in San Juan.

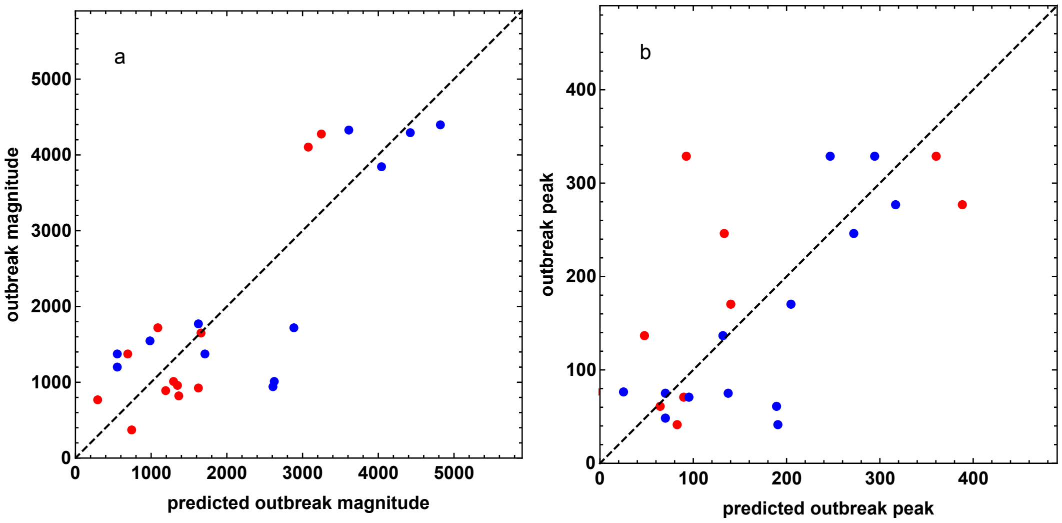

a, Predicted outbreak magnitude versus outbreak magnitude for the last 13 outbreaks in the time series of dengue hospitalizations in San Juan. The predictor variable is the mean eigenvalue $\langle \lambda_t^* \rangle$ over the assessment periods, and the relation to outbreak magnitude is trained using the first six outbreaks in the time series. The red points correspond to the assessments intervals shown in Fig. 3a, and the blue points correspond to the assessment intervals shown in Fig. 3b. **b**, Shows the same analysis as in a, but with outbreak peak instead of outbreak magnitude as the outcome variable.
